# Dielectric Relaxation and Equilibrium Thermodynamic Descriptors in Propanol Isomers: Radio-Frequency Measurements and openCOSMO-RS Analysis

**DOI:** 10.3390/molecules31193405

**Published:** 2026-09-24

**Authors:** Samir Azizov, Tarana Nurubeyli, Kamala Khalilova, Nazakat Kerimli, Gunay M. Iskenderova, Asim Abdulla, Gultamam Ganizade, Ijabika Sardarova, Orxan Aliyev, Sajara Nabieva, Afet Karimova, Jeyhun Guliyev

**Affiliations:** 1 Institute of Physics PLE, Ministry of Science and Education of the Republic of Azerbaijan, H. Javid Avenue, 131, Baku AZ-1073, Azerbaijan; 2 Risk Assessment and Management Research Center, Azerbaijan University of Architecture and Construction, Baku AZ-1073, Azerbaijan; 3 Department of Physics and Electronics, Khazar University, Mahsati Str. 41, Baku AZ-1096, Azerbaijan; 4 Department of Medical and Biological Physics Department, Azerbaijan Medical University, Samad Vurgun St.167, Baku AZ-1022, Azerbaijan; 5 Department of Electronics and Energy Engineering, Nakhchivan State University, A. Aliyev 11, Nakhchivan AZ-7000, Azerbaijan; 6 Department of Electrical and Electronics Engineering, Zonguldak Bülent Ecevit University, 67100 Zonguldak, Türkiye; 7 Department of Elektronics and Automation, Azerbaijan State Oil and Industry University, Azadliq Avenue, 20, Baku AZ-1010, Azerbaijan

**Keywords:** dielectric relaxation, n-propanol, isopropanol, hydrogen bonding, radio-frequency dielectric spectroscopy, activation energy, Kirkwood correlation factor, openCOSMO-RS

## Abstract

Dielectric relaxation of n-propanol and isopropanol was examined by radio-frequency measurements at seven fixed frequencies (0.3–32 MHz) over +20 to −160 °C and by openCOSMO-RS calculations. Apparent characteristic times were assigned from dielectric-loss maxima and analyzed with Arrhenius and Eyring formalisms. Nine temperature scans were available for each sample, obtained with three independent measuring capacitors and three repeat scans per capacitor. Capacitor-specific Arrhenius fits gave apparent activation energies of 21.30 ± 0.16 kJ mol^−1^ (SD; 95% CI of the mean 20.91–21.69) for n-propanol and 21.99 ± 0.16 kJ mol^−1^ (95% CI 21.60–22.38) for isopropanol. A conservative Welch comparison of the three independent-capacitor estimates gave ΔE_a_ = 0.687 kJ mol^−1^ (95% CI 0.332–1.042, *p* = 0.0058). Although this sub-kJ difference is statistically distinguishable in the cell-level analysis, it is not considered to have mechanistic significance because systematic temperature uncertainty and unresolved spectral overlap are not included in that comparison. Eyring activation enthalpies are 19.60 ± 0.15 and 20.23 ± 0.15 kJ mol^−1^ (SD), respectively. Entropy and Gibbs-energy values are reported only as apparent quantities under κ = 1. openCOSMO-RS yields liquid-to-n-hexane infinite-dilution transfer enthalpies of 30.5 and 28.0 kJ mol^−1^. These transfer quantities include hydrogen bonding and nonspecific solvation, dispersion, and packing contributions and are therefore not identified with hydrogen-bond enthalpies. Literature calorimetry gives lower neat-alcohol hydrogen-bonding enthalpy magnitudes of approximately 17 kJ mol^−1^. Kirkwood factors above unity indicate strong static orientational correlation. Because the seven-frequency window does not independently resolve the Debye and structural α processes, the kinetic parameters are interpreted as effective descriptors of the dominant dielectric-loss process rather than mode-specific barriers.

## 1. Introduction

Dielectric relaxation in monohydric alcohols reflects both local molecular motion and collective polarization correlations associated with transient hydrogen-bonded structures [1,2,3,4,5,6,7,8,9,10]. Broadband studies show that a slow Debye-like contribution and a faster structural α-relaxation can coexist, and that hydrogen-bond exchange, chain rearrangement, and long-ranged dipolar cross-correlations need not share a single microscopic time scale [4,7,8,9,10]. Consequently, an activation energy derived from a dielectric relaxation time is an effective kinetic descriptor rather than the dissociation enthalpy of one specified O–H···O bond.

n-Propanol and isopropanol provide a useful structural comparison, because they have the same molecular formula and hydroxyl functionality but differ in molecular geometry and packing. Previous dielectric and structural studies show that branching can alter hydrogen-bond organization and relaxation behavior [11,12,13,14,15]; however, small differences between isomers require quantitative uncertainty analysis before they can be assigned mechanistic significance.

The present measurements cover seven fixed frequencies between 0.3 and 32 MHz over +20 to −160 °C. Relaxation times are inferred from the temperatures of the dielectric-loss maxima rather than from continuous broadband spectral fits. The resulting parameters therefore characterize the dominant loss process sampled by this radio-frequency window. They do not provide an independent decomposition of the Debye and α contributions.

To provide an equilibrium thermodynamic comparison, the dielectric measurements are combined with an open-source ORCA/openCOSMO-RS workflow [16,17,18,19,20]. COSMO-RS supplies molecular-surface descriptors and liquid-to-alkane transfer thermodynamics, but the calculated transfer enthalpy is not a direct hydrogen-bond enthalpy because nonspecific dispersion, packing, cavity, and alcohol–alkane interactions also contribute. This distinction can be assessed against available calorimetric hydrogen bonding and solvation/transfer data for aliphatic alcohols [21,22]. Although experimental transfer and hydrogen-bonding enthalpies are available, openCOSMO-RS is retained because it provides a common, internally consistent framework for both isomers, including temperature-dependent transfer descriptors, σ-profiles, and σ-potentials. The calorimetric literature is therefore used to benchmark the calculations rather than being replaced by them.

The objective of this work is therefore to compare, without assuming equivalence, (i) the apparent activation scale of the dominant dielectric-loss process, (ii) static orientational correlation quantified by the Kirkwood factor, and (iii) equilibrium molecular-surface and transfer descriptors from openCOSMO-RS. Particular emphasis is placed on the limitations imposed by the restricted frequency window, the uncertainty of the small inter-isomer activation-energy difference, and the different physical meanings of kinetic and equilibrium quantities.

This formulation uses the computational results as an independent thermodynamic context rather than as a surrogate measurement of hydrogen-bond strength. Numerical similarity between energy scales is treated descriptively and is not used by itself as evidence for a unique relaxation mechanism.

## 2. Materials and Methods

### 2.1. Materials and Sample Preparation

High-purity n-propanol and isopropanol (≥99.9%, analytical grade; Sigma-Aldrich, St. Louis, MO, USA) were used as received before the additional purification described below. The isomers were selected because they retain the same molecular formula and hydroxyl functionality while differing in molecular geometry.

Before dielectric measurements, the liquids were dried and further purified by fractional distillation using a rectification column, then stored in hermetically sealed glass containers to limit subsequent uptake of atmospheric moisture and contamination.

Drying efficiency and residual water content were not quantified independently by Karl Fischer titration or another water-specific method. Because trace water can modify both the dielectric response and hydrogen-bond organization of alcohols [13,23], this is treated as a systematic limitation, particularly for interpretation of small inter-isomer differences.

### 2.2. Radio-Frequency Dielectric Spectroscopy, Data Analysis, and Repeatability Assessment

Dielectric measurements were performed by the impedance transformation method using a Keysight E4990A precision impedance analyzer (Keysight Technologies, Inc., Santa Rosa, CA, USA) (instrumental range 20 Hz–120 MHz). The present analysis uses seven fixed frequencies: 0.3, 0.8, 2.0, 5.0, 9.0, 20, and 32 MHz, with temperature scans from +20 to −160 °C. The protocol is therefore a radio-frequency temperature-scan measurement rather than a continuous broadband frequency sweep [24,25,26,27,28,29].

The measurements were based on the direct determination of the capacitance C*_x_* and the dissipation factor D of the measuring cell at each fixed frequency. The complex dielectric permittivity was expressed as ε* = ε′ − iε″. The real part of the dielectric permittivity was determined from the capacitance ratio ε′ = C*_x_*/C_0_, where C_0_ is the capacitance of the empty cell, whereas the dielectric loss was obtained from the dissipation factor as ε″ = ε′·D. The guarded electrode configuration of the measurement cells, combined with the built-in open/short/load calibration routines of the analyzer, substantially reduced the influence of connecting cables and stray capacitances on the measuring circuit, thereby providing high sensitivity for detecting small variations in the complex dielectric permittivity [27,29].

For each sample, nine temperature scans were obtained using three independent measuring capacitors, with three repeat scans per capacitor (Rep 1–3, capacitor 1; Rep 4–6, capacitor 2; Rep 7–9, capacitor 3). At each fixed frequency, the loss-peak temperature T0 was determined separately from each scan. Because τapp = 1/(2πf0) is fixed by the selected frequency, repeatability enters the Arrhenius analysis through T0 (and therefore 100/T0), not through an independently varying τapp. Across the nine scans, the pointwise SD of T0 was 0.683–0.687 K for n-propanol and 0.684–0.686 K for isopropanol (SE ≈ 0.228 K; corresponding 95% CI half-width ≈ 0.53 K). To avoid pseudoreplication, the three repeat T0 values within each capacitor were averaged at each frequency, yielding one seven-point Arrhenius dataset per independent capacitor. Three capacitor-specific Ea estimates were therefore obtained for each isomer and summarized as the mean, SD, SE, and a two-sided 95% confidence interval of the mean using the Student’s t distribution with 2 degrees of freedom. The same nested procedure was used for the Eyring analysis. A conservative unpaired Welch comparison of the three capacitor-specific Ea estimates was used for the inter-isomer difference. A full propagated uncertainty for gK remains unavailable because replicate-level ε0 values and complete uncertainties for density, optical refractive index, and dipole moment are not available; gK is therefore used qualitatively rather than for significance testing between the isomers. For each seven-point Arrhenius regression, the ordinary least-squares slope standard error and two-sided 95% confidence interval were calculated using the Student t distribution with 5 residual degrees of freedom.

At each fixed frequency *f*_0_, an apparent characteristic time was assigned from the temperature *T*_0_ of the ε″ maximum using 2π*f*_0_*τ*_app_ ≈ 1. The resulting *τ*_app_(T) relation contains seven points and is not obtained from global fitting of ε*(f) spectra. Accordingly, *τ*_app_ and the activation parameters derived from it are effective descriptors of the dominant loss process within the investigated window; they cannot be assigned uniquely to a pure Debye or α process. The cooling rate was not documented quantitatively in the archived experimental record, and no dedicated reheating hysteresis protocol or independent DSC verification was performed. Accordingly, crystallization-related perturbations cannot be excluded completely in the deepest supercooled region.

### 2.3. Quantum-Chemical Calculations and COSMO-RS Modeling

The molecular structures were generated from SMILES representations by the distance geometry method implemented in RDKit, following the openCOSMO-RS conformational protocol [16]. Preliminary geometry optimization was performed with GFN2-xTB, followed by refinement at the DFT/BP86/def2-TZVP(-f) level using the COSMO continuum model.

For each selected conformer, single-point quantum-chemical calculations were performed at the BP86/def2-TZVPD level within the CPCM continuum solvation model in order to obtain the distributions of the screening surface charges. All calculations were performed using the ORCA package, version 6.1.1, with the element-specific CPCM radii included in the openCOSMO-RS parameterization.

The thermodynamic characteristics were calculated with the open-source openCOSMO-RS implementation in Python (openCOSMO-RS version 3.11) using the openCOSMO-RS 24a parameterization, which reproduces solvation free energies and logarithms of infinite-dilution activity coefficients with reported mean deviations of approximately 0.5 logarithmic units [15,30].

The σ-profiles of the molecules were obtained by clustering the surface segments calculated within the CPCM model. The σ-potential of each individual alcohol was determined from the surface-charge distribution followed by averaging over discrete intervals of the surface screening-charge density σ according to the following expression:

*μ^S^(σ)=RTlnΓ(σ)* (1)

In this expression, *μ^S^*(σ) is the σ-potential of the surface at a given surface screening-charge density σ; Γ(σ) is the surface activity coefficient, characterizing the thermodynamic probability of the existence of surface segments with a given value of σ; R is the universal gas constant; and T is the absolute temperature of the calculation. The resulting dependence *μ^S^*(σ) characterizes the distribution of the donor–acceptor properties of the molecular surface and is used to analyze specific intermolecular interactions in liquid systems.

To calculate a model-based liquid-to-alkane transfer descriptor, the logarithm of the infinite-dilution activity coefficient, ln *γ*^∞^, of each alcohol in n-hexane was evaluated over 210–300 K [31]. Transfer from the associated neat alcohol to infinite dilution in a non-hydrogen-bonding solvent suppresses alcohol–alcohol connectivity, but the resulting thermodynamic change also contains nonspecific solvent, dispersion, cavity, and packing contributions.

Δ*H*_tr_^∞^ = R [d ln *γ*^∞^/d(1/T)]P = 1000 R [d ln *γ*^∞^/d(1000/T)]P (2)

Here, Δ*H*_tr_^∞^ denotes the openCOSMO-RS infinite-dilution liquid-to-n-hexane transfer enthalpy, *γ*^∞^ is the infinite-dilution activity coefficient, R is the gas constant, and T is absolute temperature. Because the van’t Hoff plots are expressed as 1000/T, the fitted slope must be multiplied by 1000 R. Δ*H*_tr_^∞^ is not interpreted as the enthalpy of a single hydrogen bond or as a direct hydrogen-bonding enthalpy of the neat liquid.

*H*^E^ = −RT^2^ [∂(G^E^/RT)/∂T]P,x = −RT^2^ Σ_i_ x_i_ [∂ ln γ_i_/∂T]P,x (3)

or, in finite-difference form,

*H*^E^ ≈ −RT^2^ Σ_i_ x_i_ {ln γ_i_(T + 5 K) − ln γ_i_(T − 5 K)}/(10 K) (4)

where *H*^E^ is the molar excess enthalpy of the binary mixture, *x*_i_ and *γ*_i_ are the mole fraction and activity coefficient of component *i*, *R* is the universal gas constant, *T* is the absolute temperature, and *G*^E^ is the molar excess Gibbs energy. Equation (3) emphasizes that a mixture-level excess molar enthalpy requires the composition-weighted contributions of both components; a derivative of a single ln *γ*_i_ corresponds instead to a partial molar excess quantity. The finite-difference form in Equation (4) uses a symmetric temperature interval of ±5 K and is evaluated at fixed composition and pressure.

The thermodynamic and dielectric analyses are kept conceptually separate. Δ*H*_tr_^∞^ is an equilibrium solution-thermodynamic descriptor, whereas *E*_a_ and the Eyring parameters are derived from the temperature dependence of an apparent dynamical time. Their values are compared only as distinct energetic descriptors and are also placed against experimental thermodynamic literature [21,22].

## 3. Results and Discussion

### 3.1. Temperature–Frequency Dependences of Dielectric Relaxation

Figure 1 shows the Arrhenius representation of the apparent characteristic times. At each fixed frequency f0, T0 was obtained from the ε″(T) maximum in each of the nine scans, while τapp = 1/(2πf0). For visualization, the plotted abscissa is the mean 100/T0 across the nine scans, and the horizontal error bars are ±1 SD of 100/T0. The inferential statistics reported below are based on the three independent capacitor-specific fits, with three repeat scans averaged within each capacitor.

−lg *τ*_app_ = −lg *τ*_A_ − [*E*_a_/(2.303R)](1/T) = −lg *τ*_A_ − [*E*_a_/(230.3R)](100/T) (5)

For a linear fit y = a + m(100/T), the apparent activation energy is *E*_a_ = −230.3 Rm (m < 0).

The three capacitor-specific Arrhenius fits gave Ea values of 21.146, 21.301, and 21.458 kJ mol^−1^ for n-propanol and 21.832, 21.988, and 22.146 kJ mol^−1^ for isopropanol. Thus, Ea = 21.30 ± 0.16 kJ mol^−1^ (SD; SE 0.090; 95% CI 20.91–21.69; mean R^2^ = 0.9870) for n-propanol and 21.99 ± 0.16 kJ mol^−1^ (SD; SE 0.091; 95% CI 21.60–22.38; mean R^2^ = 0.9938) for isopropanol. A conservative Welch test treating the three capacitors as the independent experimental units gave an inter-isomer difference ΔEa = 0.687 kJ mol^−1^ (95% CI 0.332–1.042, *p* = 0.0058). This statistical separation is small in absolute magnitude and does not by itself establish a distinct microscopic or steric barrier.

The −lg *τ*_app_ versus 100/T dependences are approximately linear over the temperature interval sampled by the loss maxima. The replicate-aware regression statistics and resulting apparent activation energies are summarized in Table 1.

The capacitor-specific Arrhenius regression slopes m (±regression SE; two-sided 95% CI, df = 5) were −11.043 ± 0.565 (−12.495 to −9.591), −11.124 ± 0.571 (−12.591 to −9.657), and −11.206 ± 0.577 (−12.689 to −9.724) for n-propanol, and −11.401 ± 0.400 (−12.429 to −10.374), −11.483 ± 0.404 (−12.523 to −10.443), and −11.566 ± 0.409 (−12.617 to −10.514) for isopropanol, respectively. These slope uncertainties are those of the individual seven-point ordinary least-squares fits; the between-capacitor uncertainty of the derived Ea values is summarized in Table 1.

The fitted values cover approximately 170–255 K and include deeply supercooled conditions for part of the data set. Peak-position repeatability is now quantified directly from the nine T0 determinations at each frequency. The confidence intervals in Table 1 are based on the three independent capacitor-specific activation-energy estimates and therefore account for between-cell variability without treating the three within-cell repeat scans as independent experimental units. They do not include possible systematic temperature-calibration error or uncertainty associated with unresolved overlap of dielectric processes; consequently, the sub-kJ inter-isomer difference is not used as mechanistic evidence.

For n-propanol, the present activation scale is consistent in order of magnitude with earlier dielectric studies [32,33,34]. Differences in temperature range, spectral coverage, and mode separation must nevertheless be considered when comparing absolute values across studies.

The Cole–Cole representations are close to semicircular over much of the sampled range, but the high-frequency side shows systematic deviations. These deviations are consistent with overlapping faster dispersion and demonstrate that a single-time description is only an approximation within the present frequency window.

As an example, Figure 2 shows the Cole–Cole circular diagrams for n-propanol at several temperatures.

The dielectric response is therefore described here as a dominant, correlated loss process rather than as a uniquely isolated Debye mode. Contemporary broadband work on monohydroxy alcohols distinguishes the slow Debye-like response from faster structural α-relaxation and other contributions [4,9,10,32,35].

Table 2 gives the fitted static permittivity *ε*_0_ and high-frequency limiting parameter *ε*_∞_ obtained from the circular diagrams. The fitted *ε*_∞_ values exceed the optical limit n^2^, indicating that faster dielectric dispersion remains within or beyond the high-frequency edge of the present measurement window. The corresponding temperature dependences of ε0 and ε∞ are shown in Figure 3.

Because the seven fixed frequencies do not resolve the faster mode independently, *τ*_α_ and the ratio *τ*_D_/*τ*_α_ cannot be determined reliably from these data. Continuous broadband spectra with explicit multi-process fitting would be required for a quantitative Debye/α decomposition.

Static orientational correlation was estimated with the Kirkwood–Fröhlich relation [36]:

*g*_K_ = [9 *ε*_vac_ *k*_B_ T M/(*N*_A_ ρ μ^2^)] · [(*ε*_0_ − n^2^)(2*ε*_0_ + n^2^)]/[*ε*_0_(n^2^ + 2)^2^] (6)

where *ε*_vac_ is the vacuum permittivity, n^2^ is the optical-frequency relative permittivity, μ is the gas-phase dipole moment, M is molar mass, ρ is liquid density, *k*_B_ is the Boltzmann constant, and *N*_A_ is Avogadro’s constant. Literature density values were extrapolated to the measurement temperatures. The optical n^2^ value, rather than the fitted *ε*_∞_**,** is used in the Kirkwood–Fröhlich expression because *ε*_∞_ still contains faster dielectric contributions.

The resulting *g*_K_ estimates are approximately 3.4–3.7 for n-propanol and 3.3–3.8 for isopropanol over 172–253 K. Values well above unity indicate strong static orientational correlation, but they do not identify a unique cluster topology or relaxation pathway. A formal propagated uncertainty for *g*_K_ cannot be calculated from the archived dataset because replicate-level *ε*_0_ values and complete uncertainties for ρ, *n*, and μ are unavailable. The numerical ranges are therefore interpreted qualitatively, and no statistical significance is assigned to small inter-isomer differences in *g*_K_.

The high-frequency deviation and the large Kirkwood factors jointly support correlated polarization in the liquids while also showing that the present experiment is not a complete spectral decomposition. Subsequent kinetic parameters are therefore interpreted at the level of the observed dominant loss process.

This restricted interpretation is important because local hydrogen-bond exchange, structural relaxation, and long-ranged dipolar correlations may occur on related but nonidentical time scales [7,8,9,10].

### 3.2. Eyring Parameterization of the Apparent Relaxation Rate

The apparent relaxation rate *k*_rel_ = 1/*τ*_app_ was also parameterized with transition state (Eyring) theory [37]. In its general form, the rate contains a transmission coefficient κ:

*k*_rel_ = κ(*k*_B_T/h) exp(Δ*S*_act_/R) exp[−Δ*H*_act_/(RT)] (7)

Δ*G*_act_ = Δ*H*_act_ − *T*Δ*S*_act_ (8)

ln(*k*_rel_/T) = ln κ + ln(*k*_B_/h) + Δ*S*_act_/R − Δ*H*_act_/(RT) (9)

The resulting capacitor-level Eyring parameters are summarized in Table 3.

The capacitor-specific Eyring analyses gave ΔHact values of 19.448, 19.596, and 19.747 kJ mol^−1^ for n-propanol and 20.079, 20.229, and 20.381 kJ mol^−1^ for isopropanol. The corresponding means are 19.60 ± 0.15 kJ mol^−1^ (SD; 95% CI 19.22–19.97) and 20.23 ± 0.15 kJ mol^−1^ (95% CI 19.85–20.60). Under κ = 1, the apparent activation entropies are −3.55 ± 0.35 J mol^−1^ K^−1^ (95% CI −4.40 to −2.69) and −4.36 ± 0.34 J mol^−1^ K^−1^ (95% CI −5.20 to −3.52), respectively. At 200 K, ΔGact,app is 20.31 ± 0.08 kJ mol^−1^ (95% CI 20.11–20.51) for n-propanol and 21.10 ± 0.08 kJ mol^−1^ (95% CI 20.90–21.31) for isopropanol.

For a temperature-independent transmission coefficient, Δ*H*_act_ is unaffected by κ, whereas the true activation entropy and Gibbs energy are related to the κ = 1 apparent values by ΔS‡ = Δ*S*_act,app_ − R ln κ and ΔG‡ = Δ*G*_act,app_ + RT ln κ. As an illustrative sensitivity, κ = 0.1 shifts ΔS by +19.1 J mol^−1^ K^−1^ and ΔG by −3.83 kJ mol^−1^ at 200 K; κ = 0.01 shifts them by +38.3 J mol^−1^ K^−1^ and −7.66 kJ mol^−1^, respectively. If κ varies with temperature, the fitted enthalpy can also be affected. This uncertainty precludes a unique microscopic interpretation of the activation entropy or Gibbs energy.

For an Arrhenius representation of *k*_rel_ = 1/*τ*_app_, *E*_a_ and Δ*H*_act_ are related approximately by *E*_a_ = Δ*H*_act_ + RT. Near 200 K, RT ≈ 1.66 kJ mol^−1^, consistent with the approximately 1.7–1.8 kJ mol^−1^ difference between the fitted Arrhenius and Eyring enthalpy scales.

These Eyring quantities remain kinetic descriptors of the apparent loss process and are distinct from equilibrium transfer or hydrogen-bonding enthalpies.

The n-propanol activation enthalpy is consistent in order of magnitude with previous dielectric analyses [32,33], while the present restricted spectral window should be kept in mind in cross-study comparisons.

### 3.3. σ-Profiles and Molecular Polarity

Figure 4 shows the σ-profiles of the two isomers. For both compounds, the characteristic three-region distribution of a monohydric alcohol is observed: a central alkyl-dominated region near σ ≈ 0, a donor-side region at negative σ associated mainly with the hydroxyl hydrogen, and an acceptor-side region at positive σ associated mainly with the oxygen lone-pair environment. The donor and acceptor regions are closely similar for n-propanol and isopropanol, which is consistent with comparable intrinsic hydroxyl donor–acceptor character. In contrast, the central region differs substantially; n-propanol shows a more compact alkyl-region maximum, whereas branching in isopropanol produces a more structured distribution. The σ-profiles therefore support the interpretation that the principal isomeric contrast arises from molecular geometry and packing around a chemically similar hydroxyl functionality. Within this framework, COSMO-RS helps distinguish changes in molecular-surface organization from changes in the intrinsic donor–acceptor character of the hydroxyl group; it does not by itself provide a unique microscopic decomposition of energetic and steric contributions.

### 3.4. Infinite-Dilution Transfer Enthalpy and Experimental Thermodynamic Context

Figure 5 shows the openCOSMO-RS van’t Hoff plots of ln *γ*^∞^ for each alcohol at infinite dilution in n-hexane. Their slopes define a model-derived liquid-to-n-hexane transfer enthalpy. This quantity is sensitive to disruption of the neat associated liquid, but it also includes nonspecific alcohol–alkane, dispersion, cavity, packing, and solvation contributions.

The calculated Δ*H*_tr_^∞^ values are 30.5 kJ mol^−1^ for n-propanol and 28.0 kJ mol^−1^ for isopropanol. They are therefore treated as transfer/solvation descriptors, not as direct hydrogen-bonding enthalpies.

Experimental calorimetry provides an essential reference for this interpretation. Solomonov et al. reported neat-alcohol hydrogen-bonding enthalpy magnitudes in the range 16.9–17.7 kJ mol^−1^ for aliphatic alcohols, including both propanol isomers, and liquid-to-cyclohexane transfer enthalpies for the propanols are approximately 23–24 kJ mol^−1^ [21]. Independent compilations of gas-to-alkane solvation enthalpies for 1- and 2-propanol provide additional transfer-state benchmarks [22]. These data show that the numerical value of an “association” energy depends strongly on the thermodynamic reference process.

Accordingly, the openCOSMO-RS values are useful as model-derived equilibrium descriptors and as a check on qualitative thermodynamic trends, but they should not be interpreted as a semi-quantitative measurement of hydrogen-bond strength. The dielectric activation energies (21.30–21.99 kJ mol^−1^), the experimental transfer values, and the neat-alcohol hydrogen-bonding enthalpies all lie in the broad range of tens of kJ mol^−1^, but this order-of-magnitude similarity is not mechanistically diagnostic.

Activated rotational, structural, diffusion, and viscosity processes also occur in non-hydrogen-bonding liquids, and their barriers depend on molecular size, packing, and intermolecular interactions [32]. The present dataset therefore does not permit a quantitative separation of hydrogen bonding and van der Waals/packing contributions to the apparent dielectric barrier. More specifically, related radio-frequency measurements on non-hydrogen-bonding chlorobenzene–n-hexane and iodobenzene–n-hexane systems give apparent dielectric activation energies of approximately 5.9–9.6 kJ mol^−1^, depending on composition [24]. These non-zero barriers show that activated orientational dielectric relaxation does not require hydrogen bonding. Because the molecular structures, compositions, temperature windows, and spectral regimes differ from the present propanol measurements, these values are used only as contextual reference and are not subtracted from the propanol barriers to estimate a hydrogen-bond contribution.

The capacitor-level analysis resolves a small numerical difference between the apparent activation energies: ΔEa = 0.687 kJ mol^−1^ (Welch 95% CI 0.332–1.042, *p* = 0.0058). However, the magnitude is less than 1 kJ mol^−1^, and the comparison does not include systematic temperature-calibration uncertainty or uncertainty arising from the unresolved Debye/α overlap. Accordingly, statistical separation at the cell level is not interpreted as evidence for a distinct steric, configurational, or hydrogen-bond barrier between the isomers.

### 3.5. Temperature Evolution of the σ-Potential and the Static Permittivity

Figure 6 shows the σ-potentials of the two alcohols at 220, 250, and 298 K. Both display donor-side features characteristic of hydrogen-bonding functionality. On cooling, the donor branch becomes more negative; for n-propanol it changes from approximately −7 to −15 kJ mol^−1^. Within openCOSMO-RS, this indicates increasingly favorable donor–acceptor interactions. Experimentally, *ε*_0_ also increases on cooling (Figure 3, Table 2). These parallel trends are qualitatively compatible, but neither quantity determines the microscopic time dependence of the observed dielectric-loss process.

### 3.6. Excess Enthalpy and Thermodynamic Consistency Check

Figure 7 shows the openCOSMO-RS excess molar enthalpy *H*^E^ for alcohol–n-hexane mixtures at 298 K. Both curves are positive and reach maxima of approximately 0.95 kJ mol^−1^ for n-propanol and 1.01 kJ mol^−1^ for isopropanol, consistent with endothermic disruption of favorable alcohol–alcohol interactions on dilution. Experimental alcohol–hydrocarbon mixtures are likewise strongly nonideal [38].

Because matched experimental *H*^E^ values at the same temperature and composition are not included here, Figure 7 is used only as a qualitative thermodynamic consistency check; no quantitative validation metric is claimed.

Table 4 summarizes the principal kinetic, computational, and literature thermodynamic quantities. The table is intended to emphasize their different reference processes rather than to imply equality among them.

The comparison shows why the transfer enthalpy, hydrogen-bonding enthalpy, and dielectric activation energy must remain conceptually distinct. The present results support strong orientational association in the liquids, but they do not isolate a single energetic contribution as the rate-controlling barrier.

## 4. Limitations

The principal experimental limitation is spectral resolution. Seven fixed frequencies between 0.3 and 32 MHz track the temperature migration of the dominant loss maximum but do not provide a continuous multi-decade spectrum. Debye and α contributions cannot therefore be fitted independently, and the reported *τ*_app_, *E*_a_, and Eyring parameters are effective quantities for the observed loss process rather than mode-resolved parameters.

A second limitation concerns uncertainty and sample-state control. Peak-temperature repeatability is now quantified from nine scans at each frequency, and Arrhenius/Eyring uncertainty is summarized across three independent measuring capacitors, with the three within-capacitor scans treated as technical repeats. This addresses random repeatability and between-cell variability but does not provide a complete systematic uncertainty budget for absolute temperature calibration or spectral-mode overlap. A formal propagated uncertainty for gK is also not reported because replicate-level ε0 values and full uncertainties of ρ, n, and μ are incomplete; gK is therefore interpreted only qualitatively. Drying efficiency and residual water were not quantified by Karl Fischer titration or another water-specific assay. The cooling rate was not documented quantitatively in the archived experimental record, and no dedicated reheating hysteresis protocol or independent DSC verification was performed. Possible water and phase-state effects therefore cannot be excluded completely.

A third limitation concerns the computational comparison. openCOSMO-RS is an equilibrium thermodynamic model, and its transfer enthalpies include both specific and nonspecific interactions. Available experimental calorimetric data provide an important benchmark [21,22], but neither the calculation nor the present dielectric experiment separates hydrogen-bond, dispersion, packing, and collective-dynamical contributions quantitatively. Broadband dielectric spectra and matched experimental transfer/excess-enthalpy data would be required for a more resolved decomposition.

## 5. Conclusions

This study reports radio-frequency dielectric measurements of n-propanol and isopropanol together with openCOSMO-RS equilibrium descriptors. Using three independent measuring capacitors with three repeat scans per capacitor, the capacitor-specific Arrhenius analysis gives Ea = 21.30 ± 0.16 and 21.99 ± 0.16 kJ mol^−1^ (SD), with 95% confidence intervals of 20.91–21.69 and 21.60–22.38 kJ mol^−1^, respectively. The cell-level difference is 0.687 kJ mol^−1^ (Welch 95% CI 0.332–1.042, *p* = 0.0058), but this sub-kJ separation is not assigned mechanistic significance because systematic temperature uncertainty and unresolved spectral overlap are not represented in the statistical model. Eyring analysis gives apparent activation enthalpies of 19.60 ± 0.15 and 20.23 ± 0.15 kJ mol^−1^ (SD). Kirkwood factors well above unity support strong static orientational correlation, although their formal propagated uncertainty could not be reconstructed from the available inputs.

The calculated liquid-to-n-hexane transfer enthalpies (30.5 and 28.0 kJ mol^−1^) are distinct from experimental neat-alcohol hydrogen-bonding enthalpies of about 17 kJ mol^−1^ and contain substantial nonspecific interaction contributions. Numerical similarity among these energy scales is therefore not taken as proof of a common microscopic barrier. Because the seven-frequency measurements do not separate the Debye and α processes, the extracted kinetic parameters should be regarded as effective descriptors of the dominant radio-frequency loss process. The combined experimental and computational results are most useful as complementary constraints on association and dielectric dynamics, with broadband mode-resolved spectroscopy required for a more specific mechanistic assignment.

## Figures and Tables

**Figure 1:**
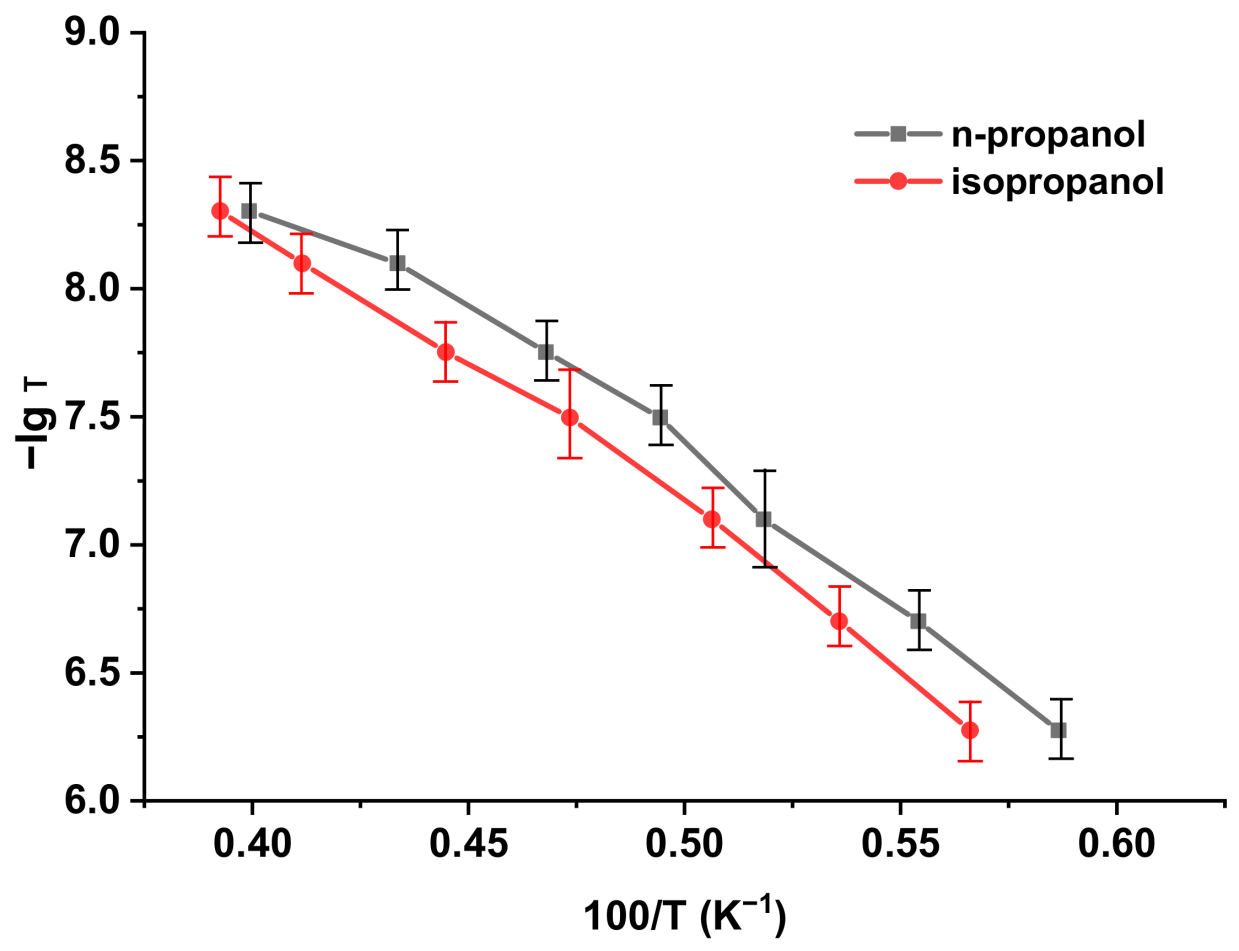
Arrhenius representation of the apparent characteristic time for n-propanol and isopropanol. The ordinate is −lg τapp and the abscissa is 100/T (K^−1^). Horizontal error bars show ±1 SD of 100/T_0_ calculated from the nine loss-peak temperatures at each frequency. The lines fit the nine-scan mean peak positions for visualization; the statistical estimates in Table 1 are based on three independent capacitor-specific fits.

**Figure 2:**
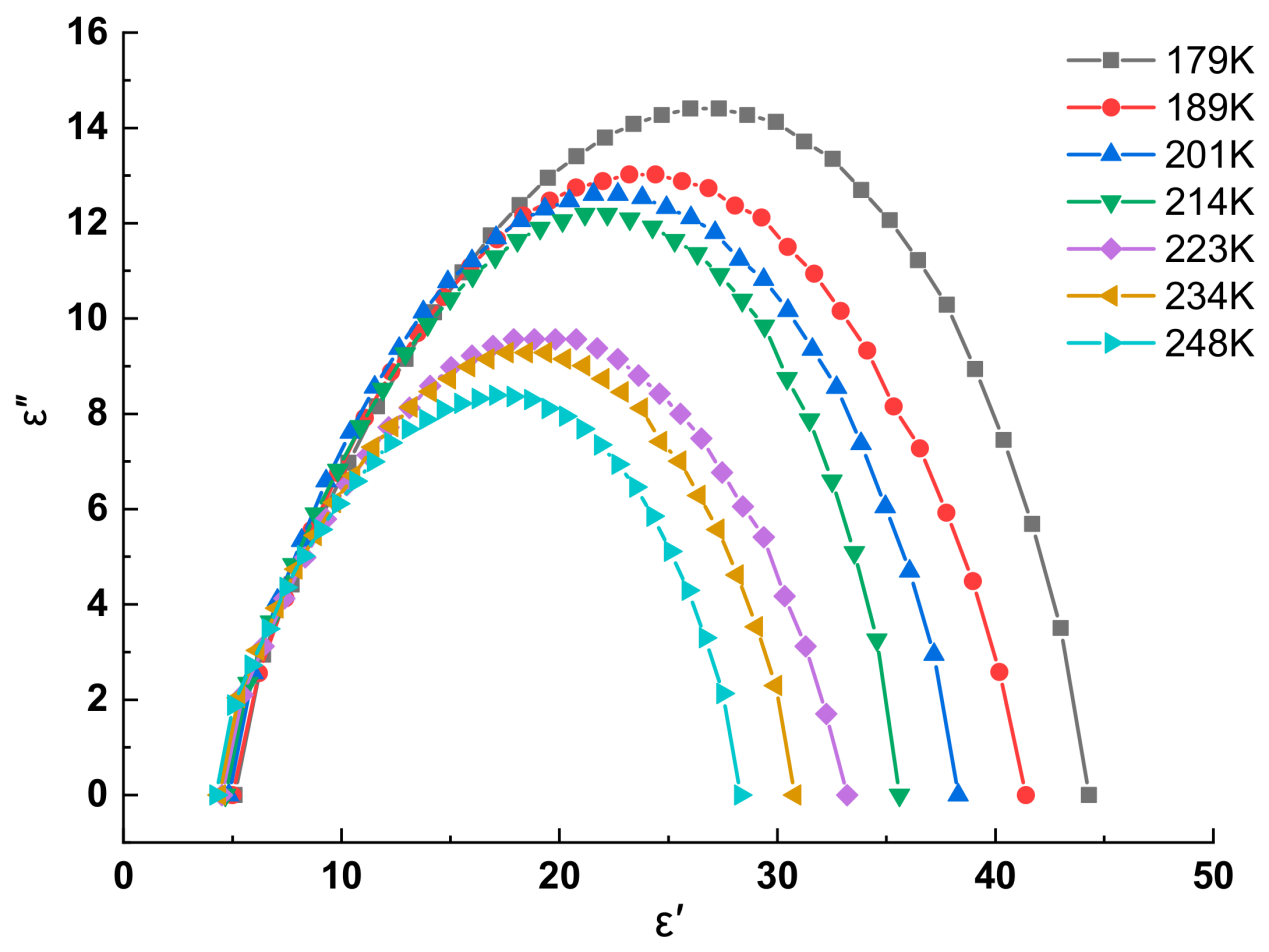
Circular diagrams (Cole–Cole diagrams) for n-propanol at various temperatures.

**Figure 3:**
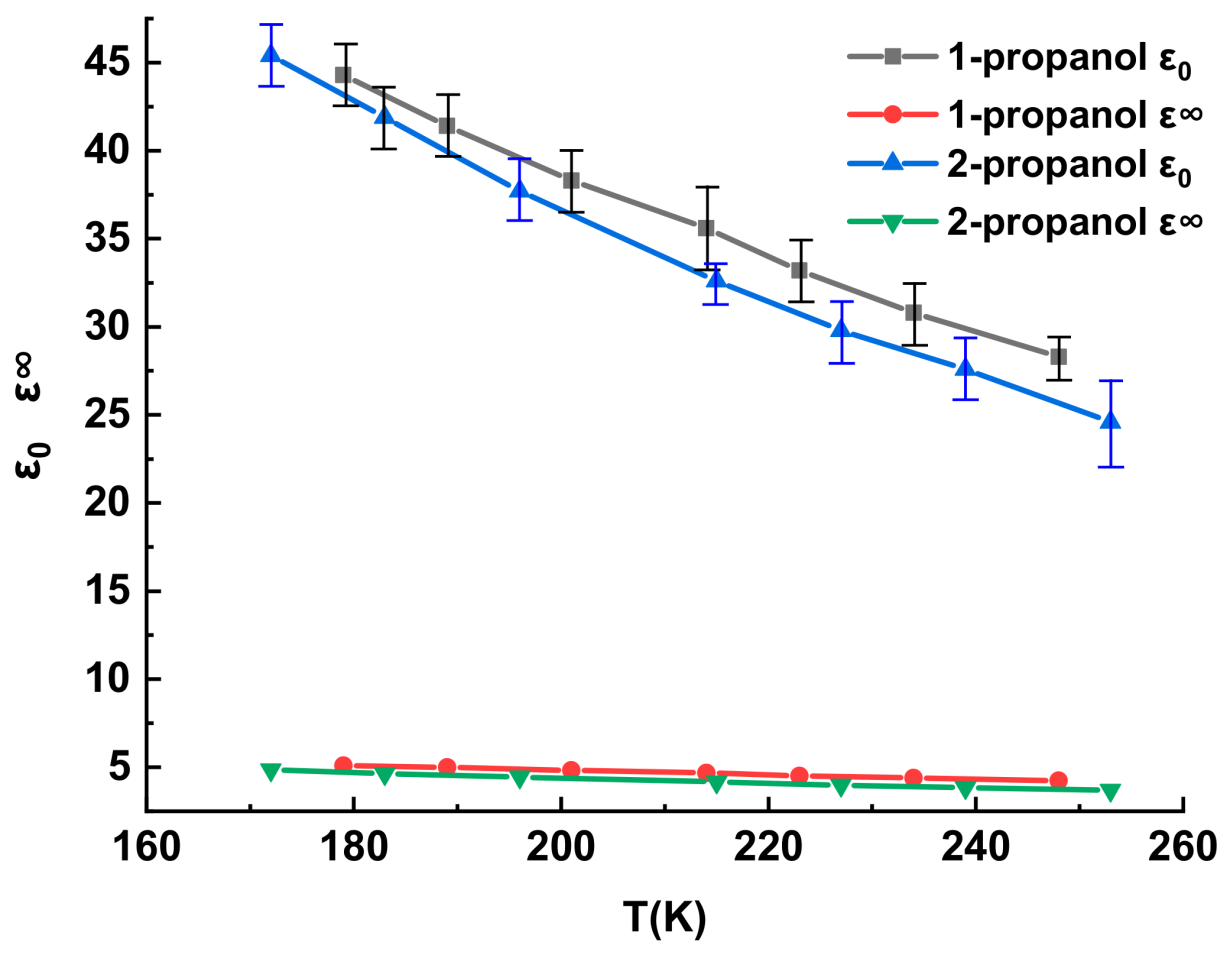
Temperature dependences of the static permittivity *ε*_0_ and the fitted high-frequency limiting parameter *ε*_∞_ for n-propanol and isopropanol, obtained from the Cole–Cole analysis. Error bars are graphical uncertainty envelopes as defined in Section 2.2.

**Figure 4:**
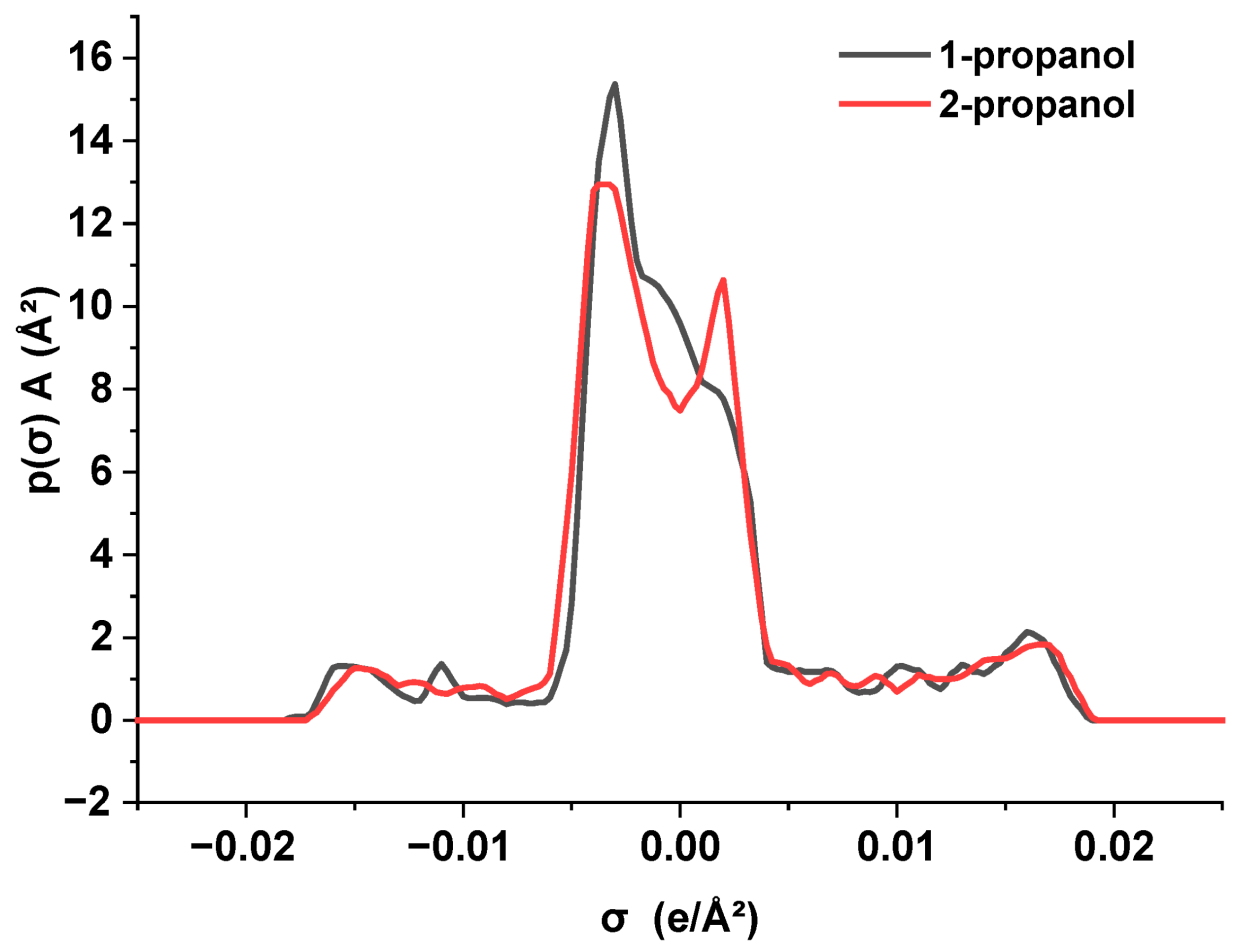
σ-profiles of n-propanol (1-propanol) and isopropanol (2-propanol) calculated using BP86/def2-TZVPD with CPCM. The dashed lines mark the adopted hydrogen-bonding thresholds at σ = ±0.0084 e Å^−2^.

**Figure 5:**
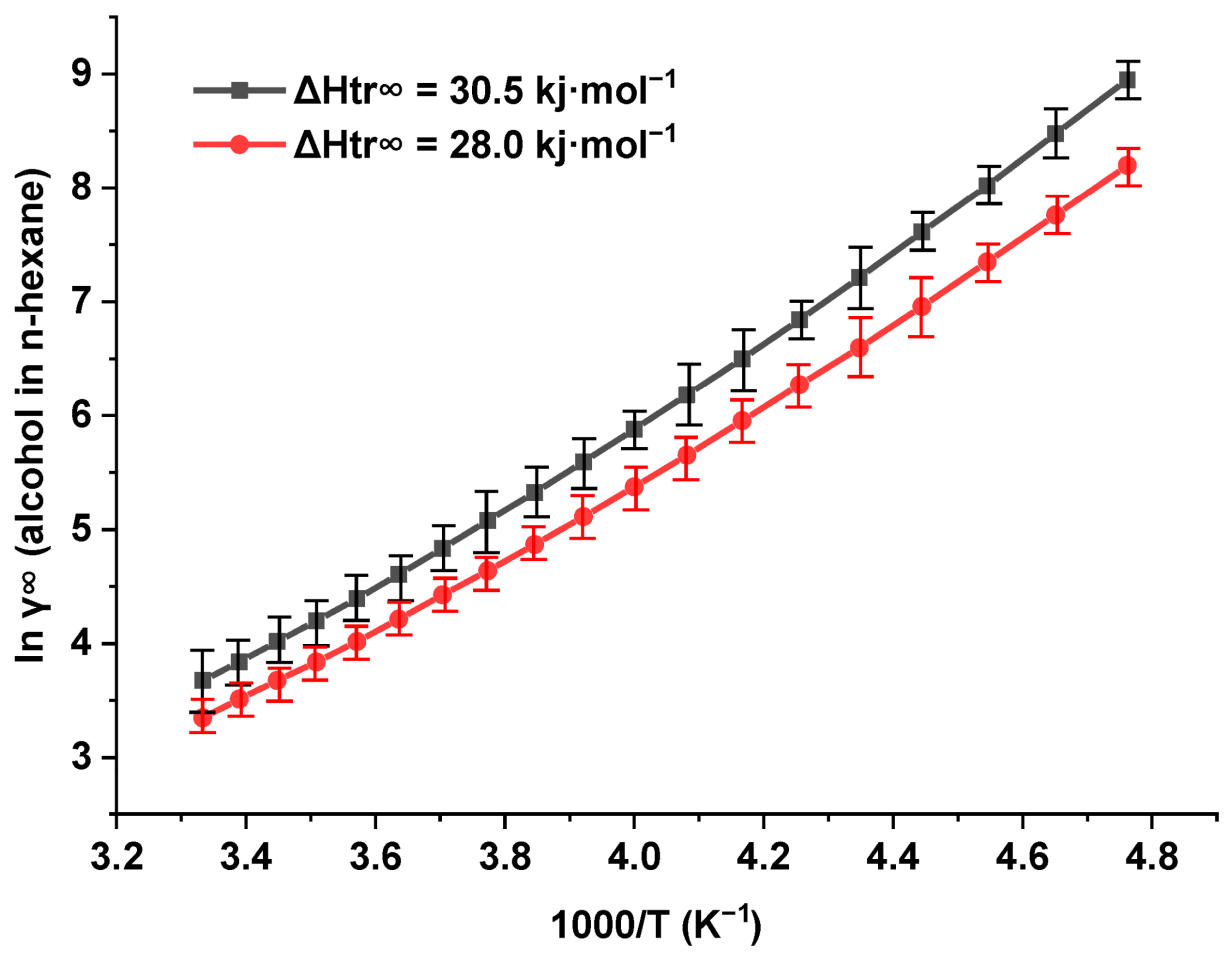
openCOSMO-RS van’t Hoff plots of ln *γ*^∞^ versus 1000/T for n-propanol and isopropanol at infinite dilution in n-hexane. The slopes correspond to Δ*H*_tr_^∞^ = 30.5 and 28.0 kJ mol^−1^, respectively.

**Figure 6:**
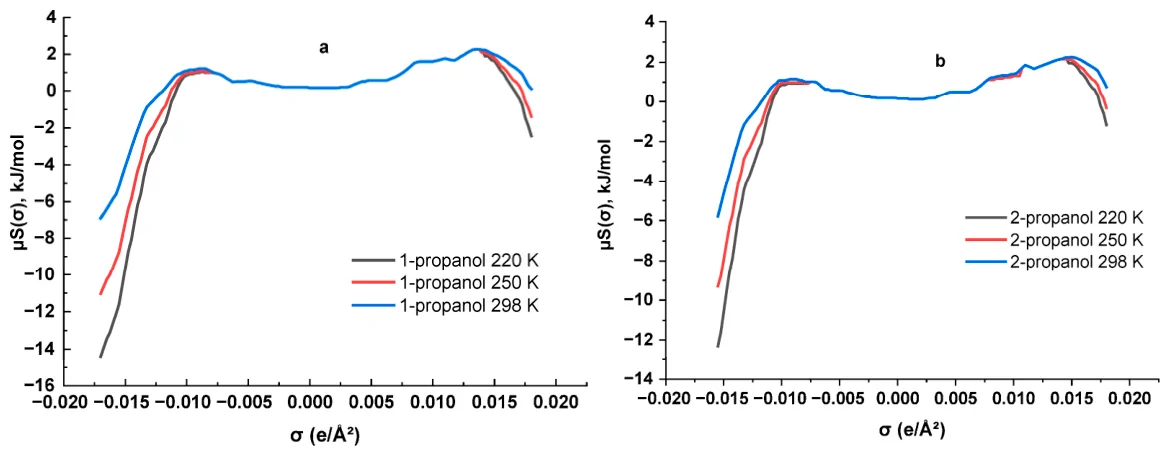
σ-potentials *μ_S_*(σ) for (**a**) n-propanol and (**b**) isopropanol at 220, 250, and 298 K. More negative donor-side values on cooling indicate increasingly favorable donor–acceptor association within the COSMO-RS framework.

**Figure 7:**
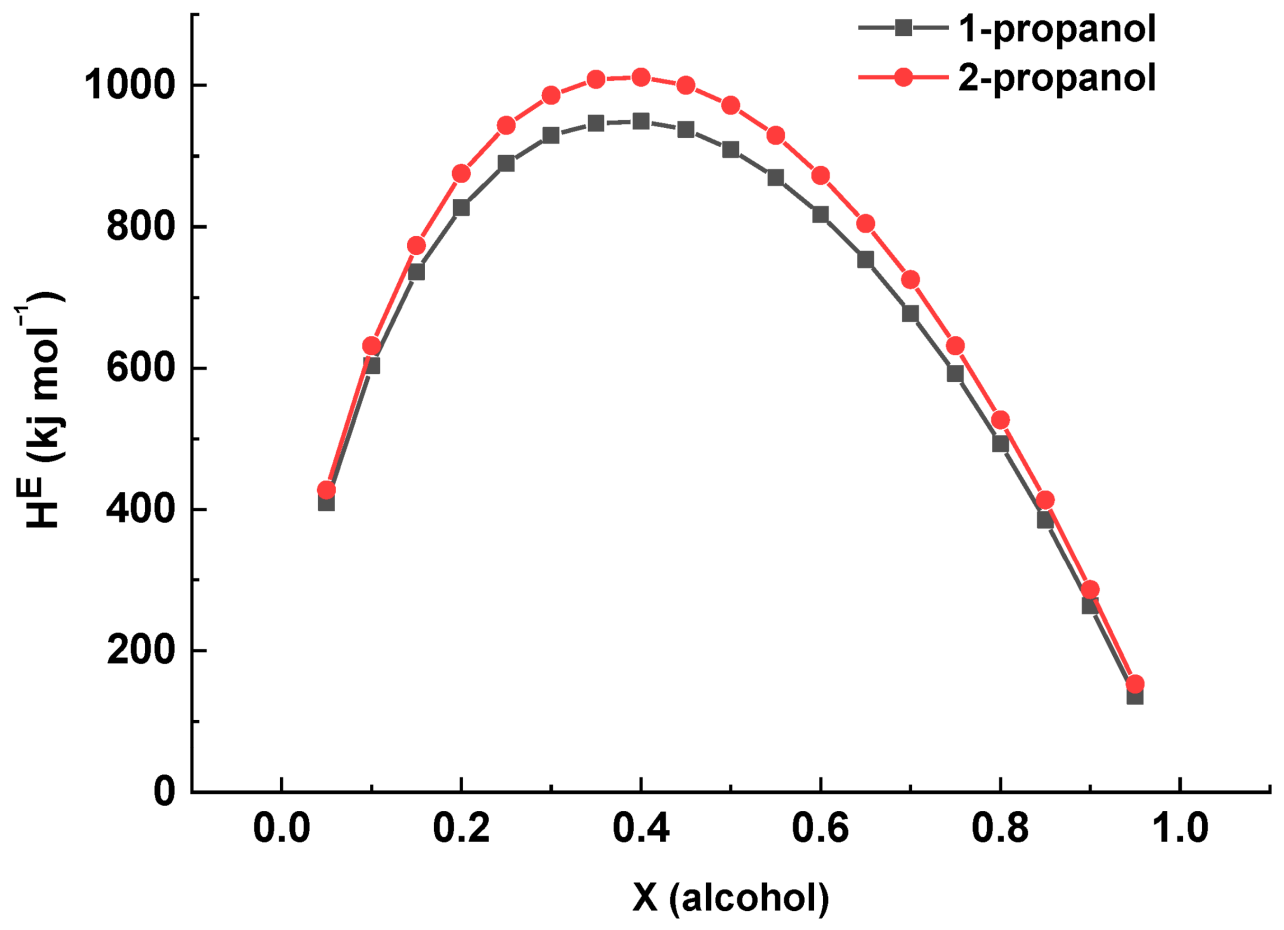
Excess molar enthalpy H^E^ of the n-propanol–n-hexane and isopropanol–n-hexane mixtures at 298 K calculated from openCOSMO-RS. The curves are model outputs and are used as a qualitative consistency check.

**Table 1:** Replicate-aware Arrhenius regression statistics for the dominant dielectric-loss process in the sampled radio-frequency window.

Alcohol	E_a_ by Capacitor (kJ mol^−1^)	Mean ± SD (kJ mol^−1^)	SE; 95% CI of Mean	Mean R^2^
n-propanol	21.146; 21.301; 21.458	21.30 ± 0.16	0.090; 20.91–21.69	0.9870
isopropanol	21.832; 21.988; 22.146	21.99 ± 0.16	0.091; 21.60–22.38	0.9938

**Table 2:** Parameters of the circular (Cole–Cole) diagrams: static permittivity *ε*_0_ and the fitted high-frequency limiting parameter *ε*_∞_ obtained for n-propanol and isopropanol at the indicated temperatures.

n-Propanol			Isopropanol		
T (K)	*ε*_0_	*ε*_∞_	T (K)	*ε*_0_	*ε*_∞_
248	28.3	4.24	253	24.6	3.70
234	30.8	4.40	239	27.6	3.85
223	33.2	4.51	227	29.8	3.98
214	35.6	4.68	215	32.6	4.17
201	38.3	4.83	196	37.7	4.45
189	41.4	5.00	183	41.9	4.64
179	44.3	5.10	172	45.4	4.85

**Table 3:** Capacitor-level Eyring results for the dominant dielectric-loss process. Values are mean ± SD across three independent measuring capacitors; 95% confidence intervals refer to the mean (df = 2). ΔSact,app and ΔGact,app are apparent quantities for κ = 1.

Alcohol	ΔHact (kJ mol^−1^)	ΔSact,app (J mol^−1^ K^−1^)	ΔGact,app at 200 K (kJ mol^−1^)	Mean R^2^
n-propanol	19.60 ± 0.15 (19.22–19.97)	−3.55 ± 0.35 (−4.40 to −2.69)	20.31 ± 0.08 (20.11–20.51)	0.9836
isopropanol	20.23 ± 0.15 (19.85–20.60)	−4.36 ± 0.34 (−5.20 to −3.52)	21.10 ± 0.08 (20.90–21.31)	0.9920

**Table 4:** Summary of principal kinetic and thermodynamic descriptors for the propanol isomers.

Parameter	n-Propanol	Isopropanol	Source/Meaning
Apparent *E*_a_ (kJ mol^−1^)	21.30 ± 0.16 (20.91–21.69)	21.99 ± 0.16 (21.60–22.38)	Experiment; mean ± SD across 3 capacitors, 95% CI
Eyring Δ*H*_act_ (kJ mol^−1^)	19.60 ± 0.15 (19.22–19.97)	20.23 ± 0.15 (19.85–20.60)	Experiment; capacitor-level Eyring mean ± SD, 95% CI
openCOSMO-RS Δ*H*_tr_^∞^ (kJ mol^−1^)	30.5	28.0	Model-derived liquid-to-n-hexane transfer enthalpy
Literature liquid-to-cyclohexane transfer enthalpy (kJ mol^−1^)	≈23–24	≈23–24	Experimental calorimetry [21]
Literature neat-alcohol H-bonding enthalpy magnitude (kJ mol^−1^)	16.9–17.7 range	16.9–17.7 range	Experimental calorimetry for aliphatic alcohols including both propanols [21]
σ-potential, donor wing at 220 K (kJ mol^−1^)	≈−15	≈−13	openCOSMO-RS surface descriptor

## Data Availability

The data supporting the findings of this study are available from the corresponding author upon reasonable request.
